# The improved genome of the nematode *Parapristionchus giblindavisi* provides insights into lineage-specific gene family evolution

**DOI:** 10.1093/g3journal/jkac215

**Published:** 2022-08-18

**Authors:** Waltraud Röseler, Maximilian Collenberg, Kohta Yoshida, Christa Lanz, Ralf J Sommer, Christian Rödelsperger

**Affiliations:** Department for Integrative Evolutionary Biology, Max Planck Institute for Biology, 72076 Tübingen, Germany; Department for Molecular Biology, Max Planck Institute for Biology, 72076 Tübingen, Germany; Department for Integrative Evolutionary Biology, Max Planck Institute for Biology, 72076 Tübingen, Germany; Department for Molecular Biology, Max Planck Institute for Biology, 72076 Tübingen, Germany; Department for Integrative Evolutionary Biology, Max Planck Institute for Biology, 72076 Tübingen, Germany; Department for Integrative Evolutionary Biology, Max Planck Institute for Biology, 72076 Tübingen, Germany

**Keywords:** comparative genomics, purine, sulfatase, metabolism, duplication, *Caenorhabditis elegans*, *Pristionchus pacificus*

## Abstract

Nematodes such as *Caenorhabditis elegans* and *Pristionchus pacificus* are extremely successful model organisms for comparative biology. Several studies have shown that phenotypic novelty but also conserved processes are controlled by taxon-restricted genes. To trace back the evolution of such new or rapidly evolving genes, a robust phylogenomic framework is indispensable. Here, we present an improved version of the genome of *Parapristionchus giblindavisi* which is the only known member of the sister group of *Pristionchus*. Relative to the previous short-read assembly, the new genome is based on long reads and displays higher levels of contiguity, completeness, and correctness. Specifically, the number of contigs dropped from over 7,303 to 735 resulting in an N50 increase from 112 to 791 kb. We made use of the new genome to revisit the evolution of multiple gene families. This revealed *Pristionchus*-specific expansions of several environmentally responsive gene families and a *Pristionchus*-specific loss of the de novo purine biosynthesis pathway. Focusing on the evolution of sulfatases and sulfotransferases, which control the mouth form plasticity in *P. pacificus*, reveals differences in copy number and genomic configurations between the genera *Pristionchus* and *Parapristionchus*. Altogether, this demonstrates the utility of the *P. giblindavisi* genome to date and polarizes lineage-specific patterns.

## Teaser

The nematode *Pristionchus pacificus* is a well established model organism for evolutionary biology. Here, we present the improved genome of its relative *Parapristionchus giblindavisi* which is the only known member of the sister group of *Pristionchus*. In comparison to the previous *P. giblindavisi* assembly, the new assembly exhibits higher levels of completeness, contiguity, and correctness. We further demonstrate the utility of the improved *P. giblindavisi* genome to date and polarizes lineage-specific patterns during nematode genome evolution.

## Introduction

The nematode *Pristionchus pacificus* had initially been introduced as a satellite model organism for comparative studies with the classical model organism *Caenorhabditis elegans* (Sommer 2006). This has extended our understanding about the evolution of single genes (Markov *et al.* 2016; Moreno *et al.* 2016), developmental networks (Casasa *et al.* 2021; Sun *et al.* 2021), and organismal phenotypes such as morphology (Rudel *et al.* 2005; Harry *et al.* 2022) and behavior (Wilecki *et al.* 2015; Moreno *et al.* 2016). More recently, *P. pacificus* developed into a stand-alone model organism to study the genetics of phenotypic plasticity (Sommer *et al.* 2017) and the origin of new genes (Rödelsperger *et al.* 2019). *Caenorhabditis elegans* and *P. pacificus* belong to 2 different nematode families of the order Rhabditida. Both species have been estimated to have diverged 130–310 million years ago (Howard *et al.* 2022). The diplogastrid family has more than 30 genera (Susoy *et al.* 2015) and includes the sister genera *Pristionchus* and *Parapristionchus*. While almost 50 species of the genus *Pristionchus* are known largely due to their common association with beetles and other insects (Kanzaki *et al.* 2021), the genus *Parapristionchus* currently has only a single member, *Parapristionchus giblindavisi* (Kanzaki *et al.* 2012). Also, only a single strain of *P. giblindavisi* was sampled from a beetle specimen and all subsequent attempts to isolate additional strains and species remained unsuccessful. Nonetheless, *P. giblindavisi* displays a number of morphological features that distinguish it from *Pristionchus* nematodes. Furthermore, phylogenetic analysis revealed deep divergence from other genera but also supports monophyly with the genus *Pristionchus* (Kanzaki *et al.* 2012). The genome of *P. giblindavisi* was sequenced as part of a phylogenomic study that involved the genomes of 8 *Pristionchus* species and 2 outgroup species to investigate the origin and evolution of new genes (Prabh *et al.* 2018). Compared to the other diplogastrid genomes in this phylogenomic data set, the assembly showed the lowest degree of contiguity and completeness level as assessed by the benchmarking of universal single-copy orthologs (BUSCO) approach (Simão *et al.* 2015). The assembly also contained the highest fraction of ambiguous base calls that could result from remaining heterozygosity or from overcompression of repeats during the assembly of Illumina short reads (Barrière *et al.* 2009; Gnerre *et al.* 2011). As the genome of *P. giblindavisi* is of particular importance to polarize lineage-specific patterns in the *Pristionchus* genus, we decided to resequence its genome using single-molecule sequencing technology of the Pacific Bioscience platform. This reduced the number of contigs by a factor of 10 and leads to 7-fold increased N50 value which is a commonly used metric to assess the contiguity of a genome assembly and which is defined as the minimal contig length in the set of largest contigs that cover at least 50% of the assembly. We further make use of the improved genome to investigate multiple lineage-specific trends of gene family evolution and to ask whether these patterns are *Pristionchus*-specific or shared with its sister genus *Parapristionchus*.

## Methods

### Worm culturing and DNA sequencing

Nematodes of the inbred *P. giblindavisi* strain RS5555 were grown on nematode growth medium. Worms were washed off of 100 fully grown plates using M9 buffer and were gently pelleted by centrifugation at 1,300× g for 1 min. The pellet was washed twice in M9 before worms were frozen in liquid nitrogen and ground to a fine powder using a mortar and pestle. The powder was directly transferred into the lysis buffer from the Qiagen genomic DNA extraction kit, which was used in combination with Qiagen genomic tip columns (500/G) (Qiagen, Hamburg, Germany). The protocol was performed following the manufacturer’s instructions. All steps involving sample vortexing were replaced by sample inverting to limit unwanted DNA shearing. DNA quality and quantity were determined with a NanoDrop ND 1000 spectrometer (PeqLab, Erlangen, Germany), a Qubit 2.0 Fluorometer (Thermo Fisher Scientific, Waltham, USA), and by a Femto pulse system (Agilent, CA, USA). A total of 15 μg genomic DNA was sheared to a target fragment size of 13 kb using a Megaruptor 2 device (Diagenode, Denville, USA). A 13-kb template library was prepared using the BluePippin size-selection system according to the manufacturer's protocol (P/N 100-286-000-07, Pacific Biosciences, CA, USA). The final library was sequenced on half of an SMRT cell of a Pacific Biosciences Sequel II instrument following the Magbead loading protocol.

### Genome assembly

PacBio raw reads were filtered for circular consensus sequencing (CCS) reads with >Q20 using PacBio ccs with –min-rq 0.99 and –chunk 10 (version 4.1.1.0). The chunked bam files were merged using pbmerge (v0.23.0) resulting in 15 Gb of CCS reads which would translate into around 60X coverage of the *P. giblindavisi* genome. BAM files were converted to FASTA format using bam2fasta program (version 1.3.0) and the genome was assembled with the Falcon-unzip2 program (version 1.8.1) while applying genome_size = 170,000,000, input_type = preads, and overlap_filterin_setting = –min-idt 99.9 options (Chin *et al.* 2016). The de novo assembly was then polished by mapping the Q20 filtered CCS reads onto the primary contigs using pbmm2 (version 1.2.0) while applying the option –preset CCS. The resulting SAM files were subsequently used to polish the genomes using racon (version 1.4.10) while applying default parameters (Vaser *et al.* 2017). We identified and softmasked repeats using RepeatScout (version 1.0.5; Price *et al.* 2005) with a seed length of 14 and the minimum number of repeats of 5 and RepeatMasker (ver. 4.1.1, http://www.repeatmasker.org; accessed 2022 Jun 1). The masked assembly was processed by HaploMerger2 to build the haploid genome (Huang *et al.* 2017). For the evaluation of genome assembly quality, the BUSCO pipeline (version 3.1.0) was run in genome mode (-m genome option) against the nematode odb9 data set (*N* = 982 orthologs; Simão *et al.* 2015). Furthermore, previously generated Illumina short reads were downloaded from the European nucleotide archive (accessions: ERR2114858, ERR2114859, ERR2114860, and ERR2114861) and were aligned against both assemblies *P. giblindavisi* assemblies using the BWA mem program (version 0.7.17-r1188; Li and Durbin 2009). The resulting alignment files were merged with the samtools merge command (version 0.1.18 r982:295; Li *et al.* 2009). Alignment statistics and ambiguous base calls were generated using the samtools flagstat and mpileup commands. From the final assembly, a single contig spanning 4.7 Mb was removed after scanning for *Escherichia coli* contamination by BLASTN analysis (*e*-value < 0.00001) against the *E. coli* genome (GenBank accession: NZ_SMTC00000000.1; Urrutia *et al.* 2020).

### Gene annotation

Evidence-based gene annotations for the *P. giblindavis*i genome were generated based on protein homology and transcriptome data using the PPCAC software (version 1.0; Athanasouli *et al.* 2020; Rödelsperger 2021). Specifically, the community-curated gene annotations of *P. pacificus* (El Paco gene annotations, version 3; Athanasouli *et al.* 2020) and a mixed-stage transcriptome assembly of *P. giblindavis*i (Rödelsperger *et al.* 2018) were transferred to the new *P. giblindavis*i genome assembly by alignment with the software exonerate (version 2.2.0; Slater and Birney 2005). For each 100-bp window, one representative gene model was chosen by selecting the gene model with the longest open reading frame (minimum protein length 60 amino acids, at least 3 exons). All other alternative gene models within a 100-bp window were discarded. To assess the quality of gene annotations, the BUSCO pipeline (version 3.1.0) was run in protein mode (-m prot option) against the nematode odb9 data set (*N* = 982 orthologs; Simão *et al.* 2015). Protein-coding genes were scanned for the presence of protein domains by the hmmsearch program of the HMMER package (*e*-value cutoff option: -E 0.001, version 3.3) using the Pfam database (Pfam-A.hmm, version 3.1b2; Bateman *et al.* 1999). The hmmsearch program was run with the same settings on proteins of 9 other diplogastrid genomes (Athanasouli *et al.* 2020; Rödelsperger 2021) and on protein data from *C. elegans*, *Bursaphelenchelus xylophilus*, *Brugia malayi*, and *Trichinella spiralis* as retrieved from WormBase ParaSite (version WBPS14; Howe *et al.* 2017). For the latter data sets, only the isoform giving rise to the longest protein sequence was analyzed. The program OrthoFinder (version 2.5.2, default mode) was used to complement the protein domain analysis (Emms and Kelly 2019).

### Phylogenetic and synteny analysis

Candidate orthologs were identified based on either the protein domains or manual BLASTP searches on http://www.pristionchus.org (accessed 2022 Jun 1), Wormbase (http://www.wormbase.org; accessed 2022 Jun 1), and Wormbase ParaSite (version WBPS16; Howe *et al.* 2017). In addition to *P. giblindavisi*, *Pristionchus* (Rödelsperger 2021), *C. elegans* (*C. elegans* Sequencing Consortium 1998), and *Caenorhabditis briggsae* (Stein *et al.* 2003) protein sequences, we included data from *Haemonchus contortus* (Doyle *et al.* 2020), *Ancylostoma ceylanicum* (Schwarz *et al.* 2015), *Ascaris suum* (Jex *et al.* 2011), *B. malayi* (Foster *et al.* 2020), *B. xylophilus* (Kikuchi *et al.* 2011), *Panagrellus redivivus* (Srinivasan *et al.* 2013), and *Plectus sambesii* (Beltran *et al.* 2019) to increase taxon sampling for better phylogenetic resolution and as outgroup species. Protein sequences were aligned by the program MUSCLE (version 3.8.31; Edgar 2004) and maximum likelihood trees were reconstructed by the phangorn R package (version 3.4.4, LG substitution model with optimization of base frequencies and invariant sites, 100 bootstrap pseudoreplicates; Schliep 2011; R Core Team 2020). The phylogenetic trees were visualized with the Dendroscope software (version 3.6.3; Huson and Scornavacca 2012). Schematic species trees were defined based on phylogenomic and phylotranscriptomic analysis (Rödelsperger *et al.* 2018; Smythe *et al*. 2019). For syntenic analysis of sulfatases and NAGLU genes, we first identified candidates based on phylogenetic analysis. Subsequently, we tested for exact collinearity by inspecting their genomic neighborhood in the genome browser.

## Results and discussion

### Single-molecule sequencing drastically increases assembly contiguity

**Fig. 1.**
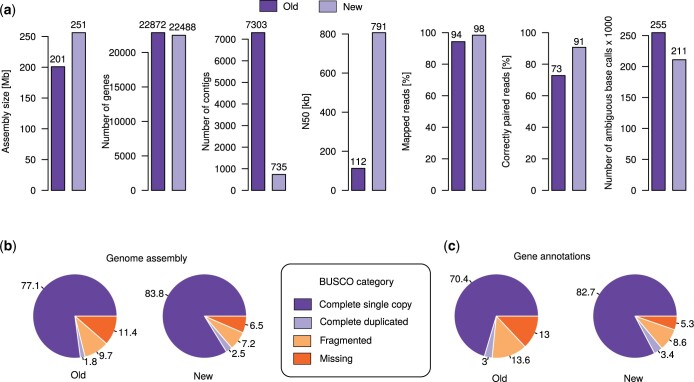
Comparison of *P. giblindavisi* genome assemblies. a) The barplots show multiple features of the old and new *P. giblindavisi* genome. b) The piecharts show the evaluation of raw genome assemblies based on the BUSCO software. c) The pie charts show the BUSCO values for the protein-coding gene annotations.

We sequenced an inbred strain of *P. giblindavisi* RS5555 on the Pacific Bioscience platform (see *Methods*). This resulted in 15 Gb of circular consensus sequences (mean length: 13 kb) which would translate into around 60X coverage of the *P. giblindavisi* genome. These data were assembled into an initial draft assembly using the FALCON-Unzip software (Chin *et al.* 2016). This assembly spanned 340 Mb that were distributed across 1851 contigs with an N50 value of 600 kb. The BUSCO completeness was 86% (complete single copy and duplicated), but showed a high level of duplicates (11%), which pointed toward assembly problems with remaining heterozygosity (Barrière *et al.* 2009). We applied the HaploMerger2 software combined with repeat masking in order to build the haploid genome (Huang *et al.* 2017). This reduced the BUSCO percentage of duplicates to 3%. The final assembly spans 256 Mb, which is 55 Mb larger than the previous version of the *P. giblindavisi* genome (Prabh *et al.* 2018). At the same time, the number of contigs was reduced from 7,303 to 735 and the N50 increased from 112 to 791 kb (Fig. 1a). To further compare the quality of both versions, we realigned the previously generated short-read data (Prabh *et al.* 2018) against both assemblies and quantified the percentage of mapped reads, pairs that were mapped in the correct orientation, and the amount of ambiguous base calls (Fig. 1a). Although these data were used to generate the initial version of the *P. giblindavisi* genome, the newly generated long-read assembly showed a much better representation of these short reads with higher fractions of mapped and correctly paired reads as well as lower fractions of ambiguous positions (Fig. 1a). These results suggest that the larger size of the new assembly is due to an overcompression of repeats in the previous assembly leading to incorrectly paired reads and higher fraction of ambiguous base calls. An assessment of genome completeness based on the BUSCO approach revealed an increase of completeness level from 79% to 86% (complete single copy and duplicated) for the raw genome assembly (Fig. 1b). Evidence-based gene annotations were generated by the PPCAC software that integrated transcriptome data from *P. giblindavisi* and protein homology with the community-curated gene annotations of *P. pacificus* (see *Methods*). This resulted in 22,488 gene models with a BUSCO completeness level of 86%, which also represents an improvement by 13% relative to the gene annotations of the previous assembly (Fig. 1c). Thus, the newly generated long-read assembly is superior to the old assembly in terms of contiguity, correctness, and completeness.

### *Pristionchus* genomes show vast expansions of gene families associated with environmental responses

**Fig. 2.**
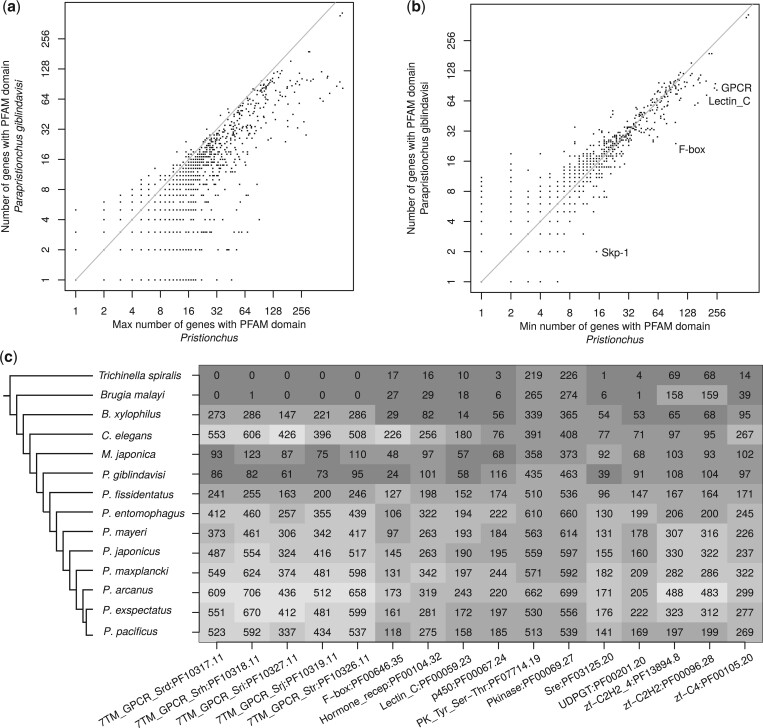
Lineage-specific expansion of large gene families. a) The scatter plot shows the number of genes with a given protein domain in the *P. giblindavisi* genome against the maximum number in any *Pristionchus* genome. The general trend toward larger gene families in *Pristionchus* is expected since a single value for a gene family in *P. giblindavisi* is compared to the maximum value from 8 *Pristionchus* species. However, the absence of strong outliers in the upper left half suggests that there are no large *Parapristionchus*-specific expansions (b) The number of *P. giblindavisi* genes with a given protein domain is plotted against the minimum number in any *Pristionchus* genome. Outliers in the lower right half indicate gene families with higher numbers in all *Pristionchus* genomes when compared to *P. giblindavisi*. c) The heatmap shows the number of genes with a given Pfam domain for 14 nematode genomes. For all shown gene families, the domain count in any *Pristionchus* genome exceeds the domain count in the *P. giblindavisi* genome by at least 50.

We made use of the improved *P. giblindavisi* genome to screen for major changes in large gene families. To this end, we compared the numbers of genes with a given protein domain in *P. giblindavisi* with the 8 available *Pristionchus* genomes (Bateman *et al.* 1999; Prabh *et al.* 2018). Specifically, we contrasted these protein domain counts in *P. giblindavisi* with the maximum count among the 8 *Pristionchus* genomes. This provides a simple method to screen for protein families that are larger in *P. giblindavisi* than in any *Pristionchus* species. However, this analysis did not reveal any large gene family expansion in *P. giblindavisi* (Fig. 2a). Hereby, we would arbitrarily define a large expansion as an increase of gene family size by at least 50 genes. A complementary analysis of orthogroups further supports the absence of large-scale expansions in *P. giblindavisi* (Supplementary Fig. 1). However, we could identify several candidates for smaller expansions such as the orthologs of the *C. elegans* glycosyltransferase *gly-7* which has one copy in most *Pristionchus* genomes but 16 copies in *P. giblindavisi* (Supplementary Fig. 1). In addition, we carried out a second screen comparing the number of protein domain counts in *P. giblindavisi* with the minimum number of the *Pristionchus* genomes. This allows us to identify candidate gene families with consistently higher domain counts in all *Pristionchus* genomes when compared to *P. giblindavisi*. Specifically, we find several candidates for large *Pristionchus*-specific expansions which include F-box genes, C-type lectins, and G-protein coupled receptors (GPCRs; Fig. 2b). To further investigate these patterns, we selected *Pristionchus* gene families with a gene family size difference of 50 or more with regard to *P. giblindavisi* and visualized the domain counts in the 10 diplogastrid genomes using the genomes of more divergent of *Micoletzkya japonica* (Prabh *et al.* 2018), *C. elegans* (*C. elegans* Sequencing Consortium 1998), *B. xylophilus* (Kikuchi *et al.* 2011), *B. malayi* (Foster *et al.* 2020), and *T. spiralis* (Mitreva *et al.* 2011) as outgroups (Fig. 2c). In addition to the above-mentioned gene families, this identified *Pristionchus*-specific expansions of nuclear hormone receptors, kinases, and zinc finger proteins. Many of these gene families integrate and respond to environmental signals. For example, C-type lectins, Cytochromes P450, and UDP-glucuronosyltransferase are thought to play key roles in detoxification of xenobiotics (Dieterich *et al.* 2008). Similarly, C-type lectins and F-box genes have been associated with immune response (Thomas 2006; Pees *et al.* 2016). Finally, GPCR and hormone receptors show enriched expression in neurons (Sural and Hobert 2021). These results point toward potential differences in the microenvironment of *Pristionchus* and *Parapristionchus* (e.g. exposure to different bacteria). However, comparative sampling efforts would be needed to experimentally test this hypothesis.

### The de novo purine biosynthesis pathway has been lost specifically in *Pristionchus*

**Fig. 3.**
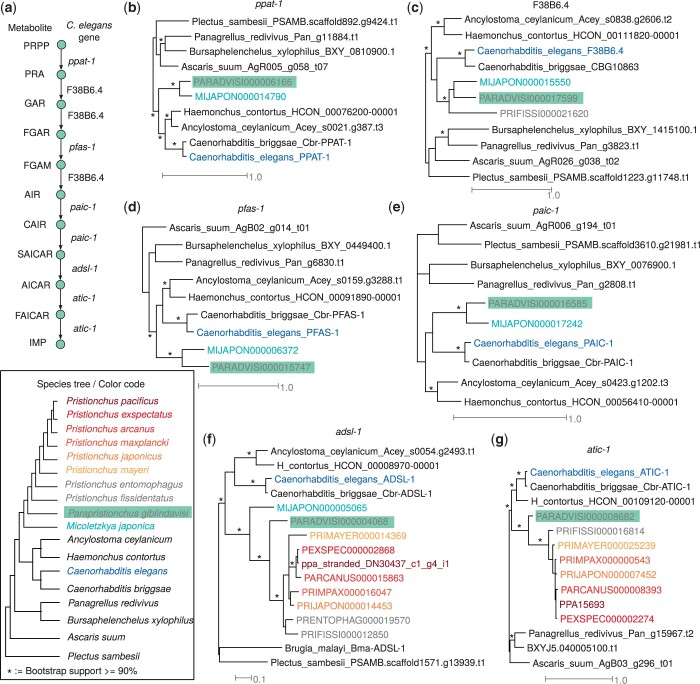
*Pristionchus*-specific loss of de novo purine biosynthesis. a) The schematic pathway shows the biosynthesis of Inosine monophosphate (IMP) from Phosphoribosyl 1-pyrophosphate (PRPP) according to the KEGG database (Ogata *et al.* 1999). Four out of 6 genes in this pathway are missing in most *Pristionchus* species. However, all these genes have orthologs in *P. giblindavisi*. This is shown by phylogenetic analysis *ppat-1* (b), F38B6.4 (c), *pfas-1* (d), *paic-1* (e), *adsl-1* (f), and *atic-1.*

In order to screen for gene families that were specifically lost in the *Pristionchus* genus, we searched for protein domains that are missing in any *Pristionchus* species but are still present in *P. giblindavisi*. This identified 15 protein domains out of which 4 domains are involved in purine metabolism (Supplementary Fig. 2). Specifically, the Pfam domains Phosphoribosylglycinamide synthetase (PF01071), AIR carboxylases (PF00731), Mur ligase (PF02875), and SAICAR synthetase (PF01259) are all associated with purine metabolism (Bateman *et al.* 1999) and are missing from all *Pristionchus* species, but are found in *P. giblindavisi*, *M. japonica*, and *C. elegans*. Previous comparative genomic analyses proposed that *P. pacificus* and also other nematodes have lost the ability to synthesize purines de novo but that the loss might be compensated by the salvage pathway that synthesizes purines from other metabolites (Desjardins *et al.* 2013). Indeed, while the first 4 enzymes of this pathway are specific to the de novo synthesis and are missing in *P. pacificus*, the last 2 enzymes encoded by the *C. elegans* genes *adsl-1* and *atic-1* also play a role in the salvage pathway and are present in *P. pacificus* (Desjardins *et al.* 2013). Our phylogenetic analysis confirms previous findings about the absence of these genes in *P. pacificus*, but adds novel data on the exact time point of this loss assigning it to the branch leading to the *Pristionchus* genus. As orthologs for all 6 enzymes could be detected in other diplogastrids such as *M. japonica* and *P. giblindavisi* the loss is specific to the genus *Pristionchus* (Fig. 3). This example demonstrates the utility of the *P. giblindavisi* genome to date lineage-specific gene losses.

### The multigene locus controlling mouth form plasticity in *P. pacificus* is not conserved outside the genus *Pristionchus*

**Fig. 4.**
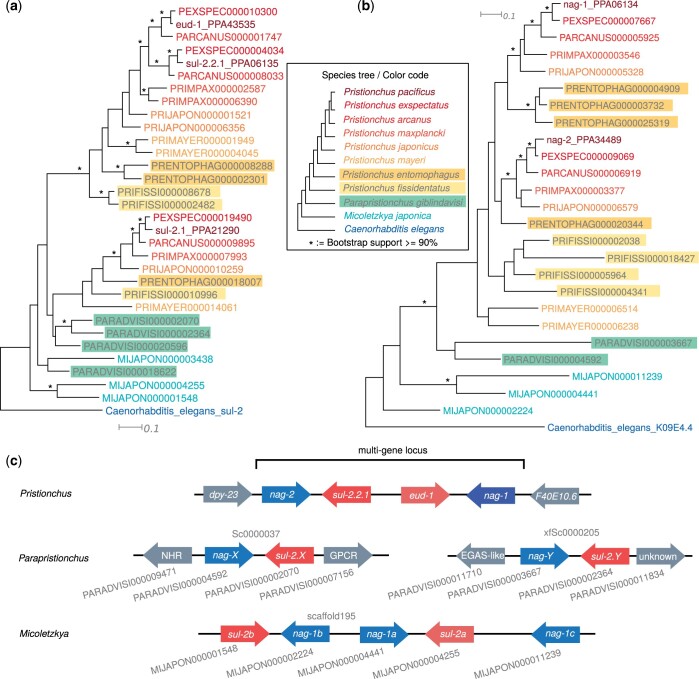
Evolution of the multigene locus controlling the mouth form plasticity. a, b) Two sulfatases and 2 *nag* genes form a multigene locus in *P. pacificus* which controls its mouth form plasticity (Sieriebriennikov *et al.* 2018). The clustering of paralogous genes in the phylogenies of sulfatases (a) and *nag* genes (b) is consistent with homogenization of paralogs by gene conversion (Sieriebriennikov *et al.* 2018). c) Syntenic analysis of candidate orthologs implies that the specific arrangement of the multigene locus in *P. pacificus* is not conserved in genomes outside the genus *Pristionchus*.

Nematodes of the diplogastrid family have evolved teeth-like structures that allow them to predate and kill other nematodes including *C. elegans* (Susoy *et al.* 2015). Simultaneously, the development of this morphological innovation had been embedded into an ancestral regulatory network controlling the plastic responses to environmental cues (Casasa *et al.* 2021). This resulted in the manifestation of 2 distinct morphs, eurystomatous and stenostomatous, of which only the eurystomatous form is capable of killing other nematodes in *P. pacificus* (Wilecki *et al.* 2015). Genetic screens in *P. pacificus* have identified multiple components of this network that are centered around the sulfatase *eud-1* (Ragsdale *et al.* 2013; Kieninger *et al.* 2016; Sieriebriennikov *et al.* 2020). This includes also the sulfotransferase *sult-1*/*seud-1* (Namdeo *et al.* 2018; Bui *et al.* 2018), which further supports that this developmental switch is regulated by sulfation processes (Igreja and Sommer 2022). The switch gene *eud-1* is part of a multigene locus that was formed by a tandem duplication of a sulfatase and a neighboring N-acetyl-alpha-glucosaminidase (NAGLU) gene (Sieriebriennikov *et al.* 2018). All existing *Pristionchus* genomes support the exact conservation of gene order of this multigene locus and phylogenetic and experimental evidence support a homogenization between paralogous gene pairs by means of gene conversion (Sieriebriennikov *et al.* 2018). While the locus had been rearranged in the genome of *M. japonica*, the genomic configuration of this locus in *P. giblindavisi* is not known. We performed phylogenetic analyses of sulfatases and NAGLUs to screen for potential orthologs in *P. giblindavisi* (Fig. 4, a and b) and tested whether these candidates are found in a syntenic region in the *P. giblindavisi* genome (Fig. 4c). This analysis revealed that the locus was split into 2 parts that reside on different contigs of *P. giblindavisi* assembly with several flanking genes at either side. These flanking genes are nonhomologous between the 2 *P. giblindavisi* loci (Fig. 4c), which supports that these 2 contigs are real and not a result of allelism (Barrière *et al.* 2009). In summary, these results suggest that the multigene locus was formed in the diplogastrid family, but was only preserved in the *Pristionchus* genus.

### *Pristionchus* and *Caenorhabditis* exhibit lineage-specific expansions in different types of sulfotransferases

**Fig. 5.**
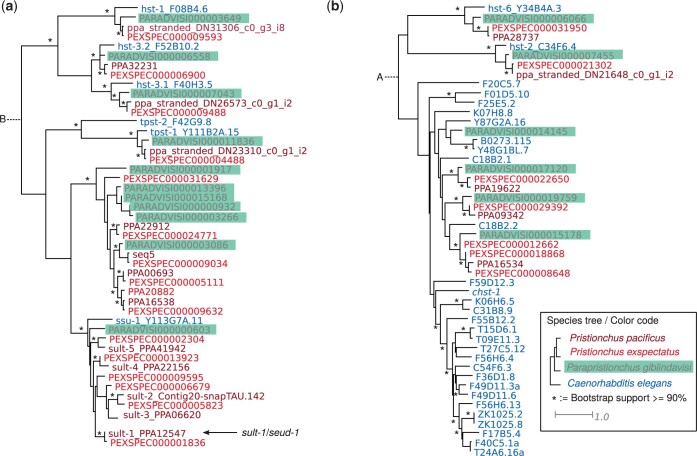
Lineage-specific expansions of sulfotransferases. a) The phylogeny shows a maximum-likelihood tree of sulfotransferases type 1 (PF00685, e.g. *ssu-1*) and 3 (PF13469, e.g. *tpst-1*). The tree is rooted with the branch leading to type 2 sulfotransferases (PF03567, e.g. *chst-1*) which are shown in (b). While cytosolic sulfotransferases (orthologs of *ssu-1*) were expanded in *Pristionchus*, type 2 sulfotransferases were expanded in *Caenorhabditis*. The *P. pacificus* gene *sult-1* that has a well-characterized role in mouth form plasticity (Bui *et al.* 2018; Namdeo *et al.* 2018) likely arose from a *Pristionchus*-specific gene expansion and has no 1-1 ortholog in *P. giblindavisi*. This is in contrast to other highly conserved sulfotransferases such *hst-1*, *hst-3.2*, *hst-3.1*, and *tpst-1*.

The sulfatase *eud-1* and sulfotransferase *sult-1* are taxonomically restricted duplicates, each of which has a single ortholog in *C. elegans* (Ragsdale *et al.* 2013; Namdeo *et al.* 2018). To date the timing of the lineage-specific expansion of *sult-1*-related sulfotransferases, we performed a phylogenetic analysis of sulfotransferases (Fig. 5, a and b). This included sulfotransferases of type 1 (PF00685, e.g. the cytosolic sulfotransferase *ssu-1*), type 2 (PF03567, e.g. the carbohydrate cytosolic sulfotransferase *chst-1*), and type 3 (PF13469, e.g. the tyrosylprotein sulfotransferase *tpst-1*) (Bateman *et al.* 1999; Igreja and Sommer 2022). In total, we found 18 sulfotransferases in *P. pacificus*. Some of these genes such as *tpst-1*, *hst-1*, *hst-2*, *hst-3.1*, *hst-3.2*, and *hst-6* have been preserved as 1-1 orthologs between all analyzed nematodes. However, different clades of sulfotransferases were expanded in the *Caenorhabditis* and *Pristionchus* lineage, respectively. Specifically, while cytosolic sulfotransferases (orthologs of *ssu-1*) are expanded in the *Pristionchus* genus (Fig. 5a), carbohydrate sulfotransferases are expanded in *C. elegans* (Fig. 5b). Notably, *sult-1* has only a single ortholog in *P. giblindavisi*, which implies that the duplication events giving rise to *sult-1* paralogs in *P. pacificus* occurred specifically within the *Pristionchus* lineage. Further phylogenetic analysis shows that all *Pristionchus* species have several *sult-1* orthologs (Supplementary Fig. 3). Thus, *sult-1* likely originated from a *Pristionchus*-specific sulfotransferase expansion.

### Conclusions

Forward genetic screens in *P. pacificus* have led to the discovery of novel genes that control development and behavior (Mayer *et al.* 2015; Lightfoot *et al.* 2019). Investigating the evolutionary history of these novel genes allows us to infer hypotheses about the evolution of associated traits. In this context, the phylogenomic framework of *Pristionchus* and other diplogastrid nematodes has been particularly helpful to date the origin of novel genes or other genomic features (Prabh *et al.* 2018; Casasa *et al.* 2021). The genome of *P. giblindavisi* has a key role in these types of analyses as it allows to answer whether genomic features are restricted to the genus *Pristionchus* or are shared with other diplogastrid nematodes. In this study, we made use of single-molecule sequencing data to generate an updated version of the *P. giblindavisi* assembly. Relative to the previously published genome (Prabh *et al.* 2018), our current assembly exhibits higher contiguity, correctness, and completeness. Furthermore, we have demonstrated multiple analyses that show how these data could be used to investigate lineage-specific patterns in *Pristionchus* nematodes. Thus, the improved *P. giblindavisi* genome lays the basis for future phylogenetic studies.

## Supplementary Material

jkac215_Supplementary_Data

## Data Availability

The genome assembly and sequencing reads have been uploaded to the European Nucleotide archive under the study accession PRJEB53331. The *P. giblindavisi* data (version 2) is also available at http://www.pristionchus.org and was submitted to WormBase ParaSite. Supplemental material is available at *G3* online.
