# Supplementary material for: The improved genome of the nematode Parapristionchus giblindavisi provides insights into lineage-specific gene family evolution
Source: G3 (Bethesda). 2022 Aug 18;12(10):jkac215. doi: 10.1093/g3journal/jkac215 (PMC9526060; doi:10.1093/g3journal/jkac215)
Supplement: jkac215_Supplementary_Data [file jkac215_supplementary_data.docx]

**Supplementary material for**

## **The improved genome of the nematode *Parapristionchus giblindavisi* provides insights into lineage-specific gene family evolution**

**Waltraud Röseler^1^, Maximilian Collenberg^2^, Kohta Yoshida^1^, Christa Lanz^2^, Ralf J. Sommer^1^, Christian Rödelsperger^1^**

Department for Integrative Evolutionary Biology, Max Planck Institute for Biology, Max-Planck-Ring 9, 72076 Tübingen, Germany

^2^ Department for Molecular Biology, Max Planck Institute for Biology, Max-Planck-Ring 9, 72076 Tübingen, Germany

^*^ Corresponding author’s email address: christian.roedelsperger@tuebingen.mpg.de


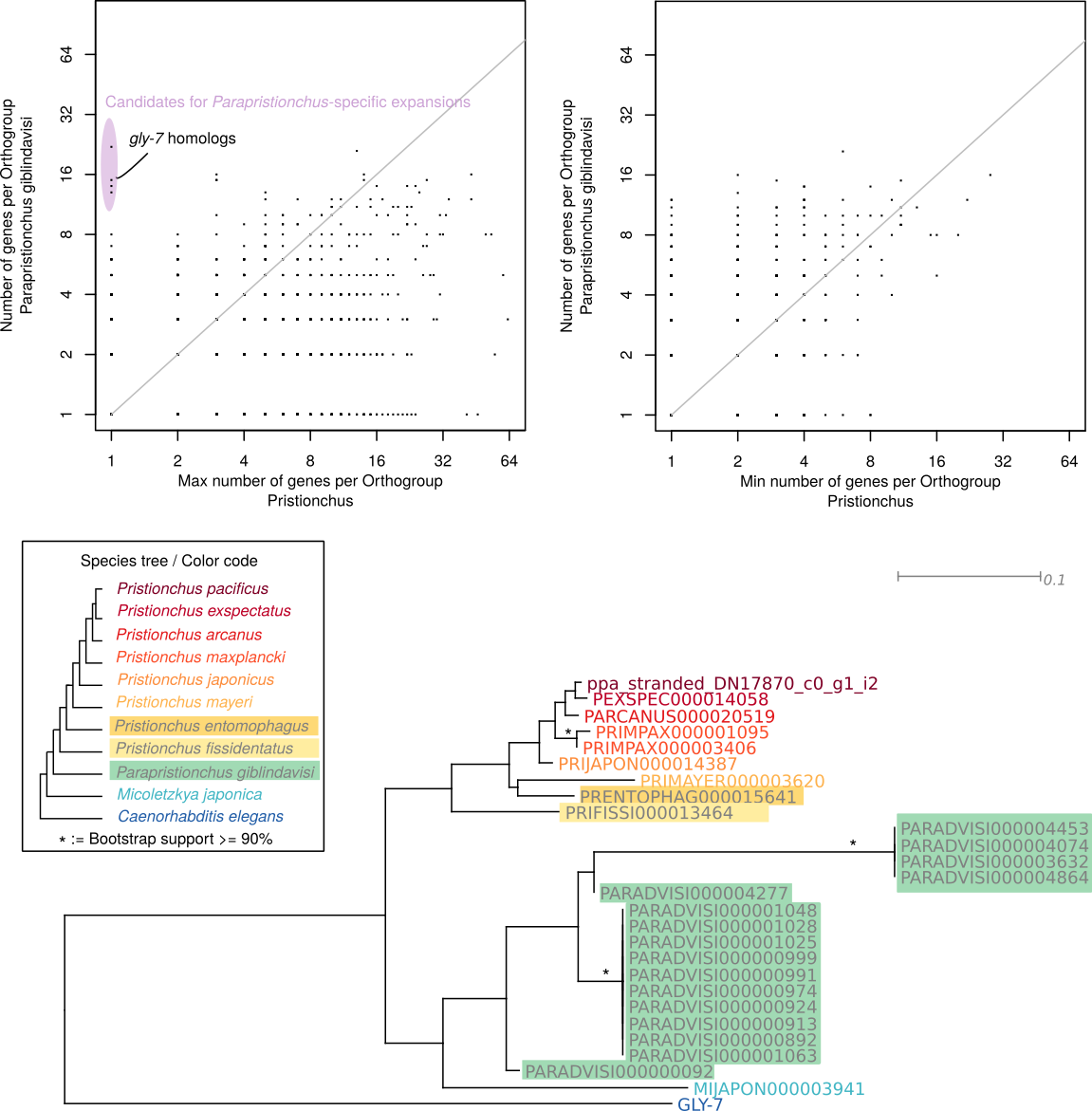


**Fig. S1 Identification of gene family size differences based on orthology clustering.** The scatter plots show the number of genes per orthogroup for the *P. giblindavisi* genome against the maximum and minimum number in any *Pristionchus* genome. We chose to investigate four candidate orthogroups in more detail that have at most one gene in *Pristionchus* but more than 10 in *P. giblindavisi*. Three of the candidate orthogroups correspond to *P. giblindavisi*-specific gene families with weak similarity in one *Pristionchus* genome (~30% sequence identity). The fourth candidate corresponds to orthologs of the *C. elegans* glycosyltransferase *gly-7*. The tree shows the phylogenetic relationships of *gly-7* orthologs including 16 copies in *P. giblindavisi*.


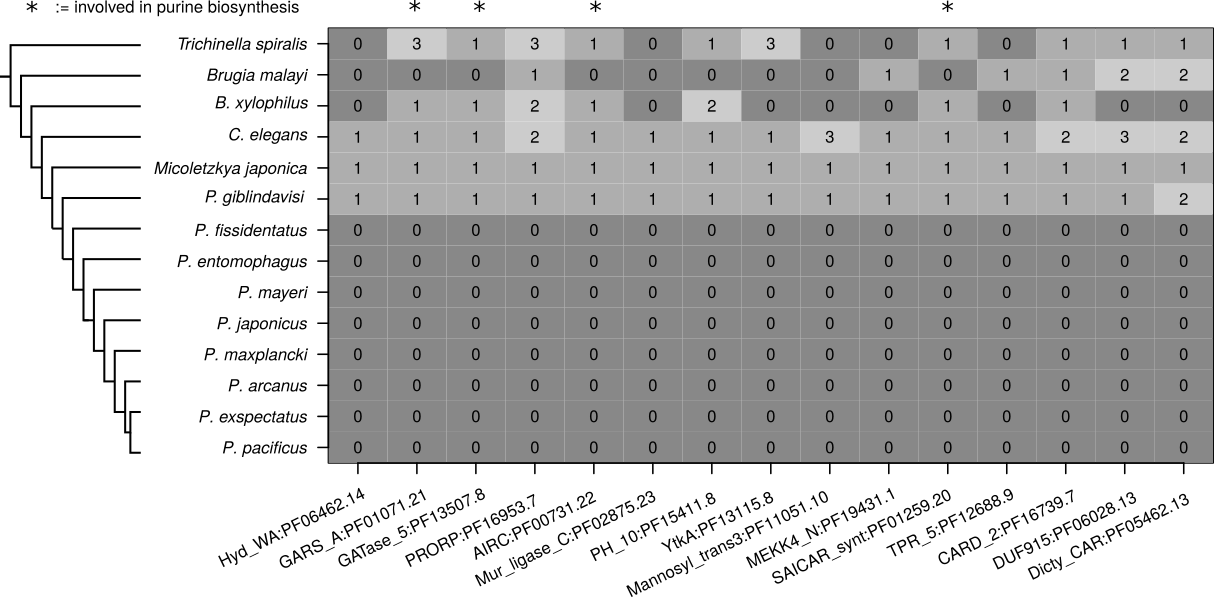


**Fig. S2 Protein domain analysis identifies candidates for *Pristionchus*-specific losses**. The heatmap shows the number of genes with a given protein domain for 15 candidate domains that exist in *P. giblindavisi* but are missing in all *Pristionchus* species. Four of these domains are involved in purine metabolism.


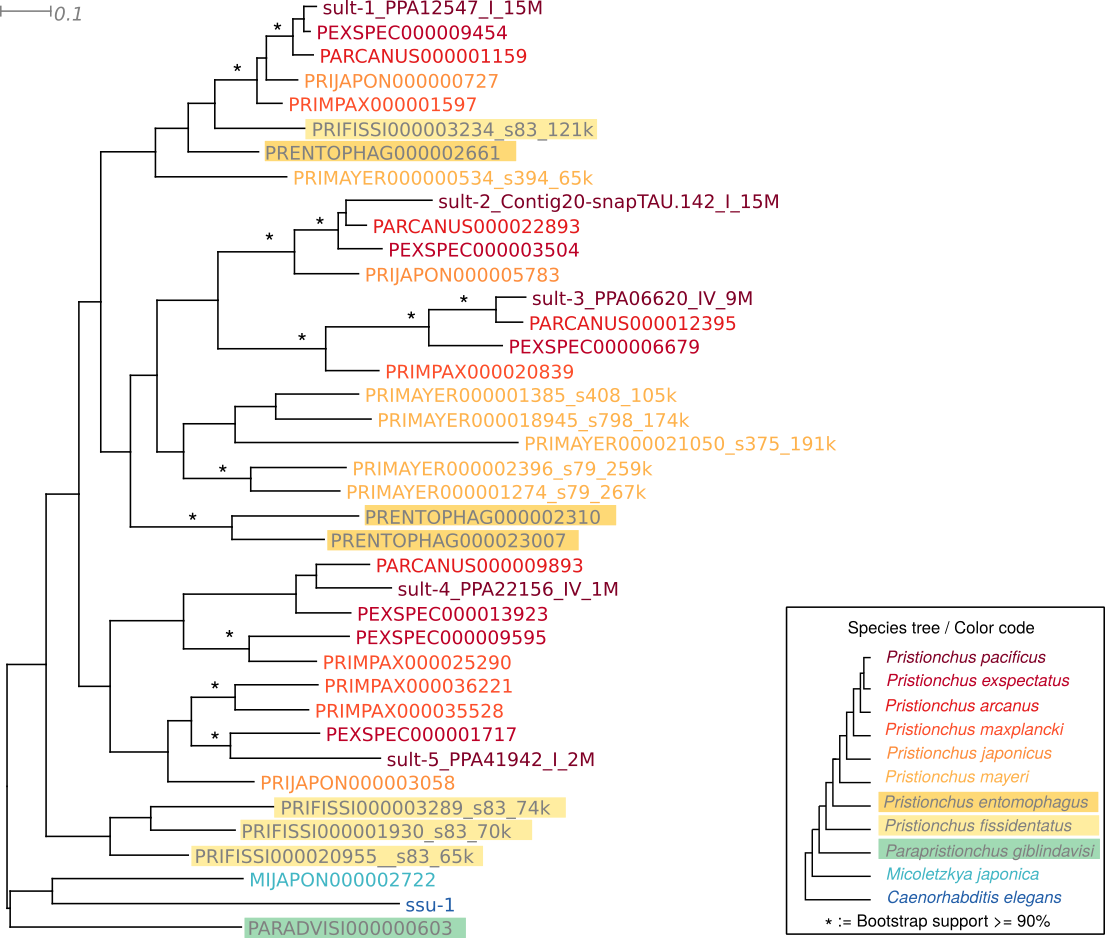


**Fig. S3** ***sult-1*/*seud-1* originated from a *Pristionchus*-specific sulfotransferase expansion.** The *C. elegans* gene *ssu-1* has multiple orthologs in *P. pacificus* and other *Pristionchus* species but only a single ortholog in the diplogastrids *M. japonica* and *P. giblindavisi*. Three *P. fissidentatus* orthologs are genomically colocalized (scaffold83, in the region 65-74kb) , which could be interpreted as a result of a recent duplication or homogenization by gene conversion.
